# Excellent Functional Outcome and Quality of Life after Primary Cementless Total Hip Arthroplasty (THA) Using an Enhanced Recovery Setup

**DOI:** 10.3390/jcm10040621

**Published:** 2021-02-06

**Authors:** Franziska Leiss, Julia Sabrina Götz, Günther Maderbacher, Matthias Meyer, Jan Reinhard, Florian Zeman, Joachim Grifka, Felix Greimel

**Affiliations:** 1 Department of Orthopedics, University Medical Center Regensburg, Asklepios Klinikum Bad Abbach, Kaiser-Karl-V.-Allee 3, 93077 Bad Abbach, Germany; f.leiss@asklepios.com (F.L.); ju.goetz@asklepios.com (J.S.G.); guenther.maderbacher@ukr.de (G.M.); ma.meyer@asklepios.com (M.M.); j.reinhard@asklepios.com (J.R.); j.grifka@asklepios.com (J.G.); 2 Center for Clinical Studies, University Medical Center Regensburg, Franz-Josef-Strauss-Allee 11, 93053 Regensburg, Germany; florian.zeman@ukr.de

**Keywords:** total hip arthroplasty, THA, ERAS, enhanced recovery, fast track, functional outcome, QoL

## Abstract

Background: Total hip arthroplasty combined with the concept of enhanced recovery is of continued worldwide interest, as it is reported to improve early functional outcome and treatment quality without increasing complications. The aim of the study was to investigate functional outcome and quality of life 4 weeks and 12 months after cementless total hip arthroplasty in combination with an enhanced recovery concept. Methods: A total of 109 patients underwent primary cementless Total Hip Arthroplasty (THA) in an enhanced recovery concept and were retrospectively analyzed. After 4 weeks and 12 months, clinical examination was analyzed regarding function, pain and satisfaction; results were evaluated using Harris Hip score, Western Ontario and McMaster Universities Osteoarthritis Index (WOMAC), EQ-5D-5L, EQ-VAS and subjective patient-related outcome measures (PROMs). Preoperatively, HADS (Hospital Anxiety and Depression Scale) was collected. A correlation analysis of age, American Society of Anesthesiologists (ASA), HADS and comorbidities (diabetes mellitus, art. hypertension, cardiovascular disease) with WOMAC, Harris Hip score (HHS) and EQ-5D was performed. Results: Patients showed a significant improvement in Harris Hip score 4 weeks and 12 months postoperatively (*p* < 0.001). WOMAC total score, subscale pain, subscale stiffness and subscale function improved significantly from preoperative to 12 months postoperative (*p* < 0.001). EQ-5D showed a significant improvement preoperative to postoperative (*p* < 0.001). The influence of anxiety or depression (HADS-A or HADS-D) on functional outcome could not be determined. There was a high patient satisfaction postoperatively, and almost 100% of patients would choose enhanced recovery surgery again. Conclusion: Cementless THA with the concept of enhanced recovery improves early clinical function and quality of life. PROMs showed a continuous improvement over a follow-up of 12 months after surgery. PROMs can help patients and surgeons to modify expectations and improve patient satisfaction.

## 1. Introduction

Total hip arthroplasty combined with the concept of enhanced recovery is of worldwide interest and in intense discussion in the recent literature [1,2,3,4,5,6]. Besides alteration of medical treatment details, the concept includes optimizing logistical and organizational aspects, implementing a multimodal opioid-sparing pain therapy and a structured management. To accelerate the recovery process in general, Kehlet et al. used evidenced-based medical interventions to minimize surgical stress, to reduce physical and psychological trauma [7]. It could be shown that an enhanced recovery concept after THA reduced the length of hospital stay with no significant increase in the incidence of complications and readmission [8,9,10,11]. Furthermore, enhanced recovery after total hip arthroplasty (THA) and total knee arthroplasty (TKA) showed no restriction for older patients or patients with comorbidities like cardiopulmonary disease or type II diabetes [12,13].

In the USA and most European countries, THAs are implanted in more than 200/100,000 inhabitants yearly, and in November 2019, 300/100,000 are reported for Germany (OECD—Organization for Economic Cooperation and Development). As the population is aging, the demand for THA will remain high, and economic pressures dictate a need for improved efficiency and a reduction of costs for the health care systems [14]. 

Major surgery like total hip arthroplasty is followed by a convalescence period, which can lead to the loss of muscle strength and function [15,16]. Early rehabilitation, like fast track after THA, with mobilization on the day of surgery, enables less catabolism and loss of postoperative muscle mass and function, improved pulmonary function, early recovery of gastrointestinal function and reduced thromboembolic complications [17,18]. After a surgical procedure, patients express concern about pain and dependence on other persons. Therefore, a key issue of the enhanced recovery concept is patient motivation and transfer of responsibility to the patient through intensive information and preoperative patient education. Multidisciplinary cooperation is therefore of great importance, to highlight all relevant aspects.

The aim of the study was to evaluate the functional outcome and quality of life 4 weeks and 12 months after cementless total hip arthroplasty in combination with a concept of enhanced recovery. Furthermore, we wanted to evaluate whether comorbidities like cardiovascular disease, arterial hypertension or diabetes type II or preoperative anxiety/depression influence outcome after fast track THA.

## 2. Materials and Methods

In the present retrospective study, 109 of 119 patients who underwent primary cementless collarless THA (DePuy Corail^®^ femoral stem, DePuy Pinnacle^®^ acetabular component, DePuy Orthopaedics, Warsaw, IN, USA) with an enhanced recovery concept in a single center were included. Inclusion criteria were elective primary THA due to primary or secondary osteoarthritis conducted between mid-2018 and mid-2019 after implementing enhanced recovery in a part of the facility. Exclusion criteria were severe dysplasia of the hip, use of other components prosthetic than those mentioned above, obesity III° (body mass index (BMI) > 40 kg/m^2^), malignancy and immobility. All data were extracted from digitized patients records.

The study was approved by the local Ethics Committee (19-1352-104, IRB approval). The study was applied in accordance with the ethical standards of the Declaration of Helsinki 1975.

The enhanced recovery program consisted of preoperative gait training with crutches and a detailed multidisciplinary lecture. Just before the procedure, a non-steroid-anti-inflammatory drug (etoricoxib 90 mg one hour before the intervention) was applicated. The operation was performed under spinal anesthesia (prilocaine 1% hyperbaric 4 mL = 80 mg, and sufentanil 10 µg and dexamethasone 8 mg i.v. as standard) by using an anterolateral approach (Microhip). Intraoperatively, tranexamic acid was administered topically (2 g) and intravenously (1 g). Local-infiltration analgesia was applied in the periacetabular and femoral region as well as subcutaneously (200 mg ropivacaine, for the deep periarticular infiltrations with 0.5 mg adrenalin). No wound drains were used. Full weight bearing was allowed right away, and the patients were mobilized for the first time 2–3 h after the operation. All patients received physiotherapeutic treatment twice a day and were instructed to use a newly established exercise circuit. The exercise circuit included a walking course, various muscle exercises and tutorials to improve coordination. Furthermore, a mirror-wall with a holding bar was installed in the ward to ensure that patients repeated exercises independently. The exercise circuit focuses on strengthening hip and knee muscles. In our department, a standardized pain management concept was established regarding the recommendations within the World Health Organization (WHO) analgesic ladder [19]. With the introduction of the enhanced recovery concept in our department, a number of scores like EQ-5D-5L, Western Ontario and McMaster Universities Osteoarthritis Index (WOMAC) and Harris Hip score (HHS) were recorded routinely. The contents of the enhanced recovery concept are shown in Table 1. We did not aim for a reduction of hospital length of stay (LOS), and the mean duration of stay was 7 days. 

General data age, sex and BMI, operated leg, ASA-score (American Society of Anesthesiologists), ECM (Elixhauser Comorbidity Method) and comorbidities like coronary heart disease, arterial hypertension or diabetes mellitus type II were assessed (Table 2). The ECM originally counts 30 unweighted variables, and, according to the literature, patients were categorized in 0, 1, 2 and greater than or equal to 3 [20].

Preoperatively, all included patients completed the Harris Hip score (HHS), the Euroqol quality of life (EQ-5D-5L), Euroqol visual analog scale (EQ-VAS) (0–100, 0 = worst possible health status, 100 = best possible health status), the Western Ontario and McMaster Universities Osteoarthritis Index (WOMAC) and the Hospital Anxiety and Depression Scale (HADS) tests as standard. The maximum value of Harris hip score is 100. Excellent results/: 90–100 points; good results: 80–89 points; moderate results: 70–79; bad result: 60–69 points. Western Ontario and McMaster Universities Osteoarthritis Index (WOMAC) is ordinal scaled on a scale from 0 to 4 points. The range of scores for the respective subscales are pain 0–20 points, stiffness 0–8 points and physical function 0–68 points. A higher point value corresponds to a worse result. EuroQol-5D-5L (EQ-5D) provides a description of five dimensions of health status. The value sets are anchored on 11111 = 1, representing full health and dead = 0. The HADS is a valid and reliable tool and has 14 questions, seven for anxiety (HADS-A) and seven for depression (HADS-D). A final score ≤7 is indicative of no anxiety or depression. A score of 8–10 is indicative of borderline anxiety or depression, and a score ≥ 11 is indicative of clinical anxiety or depression. All cases presented 4 weeks postoperatively, including a physical examination, evaluation of the HHS and additional subjective patient-related outcome measures (PROMs). Our questions were implemented to record and analyze the functional results and the quality of life of the patients. The PROM questions were the following: Was the operation successful in your eyes (yes/no)? Would you perform surgery (THA) again (yes/no)? Furthermore, the following questions were analyzed, and possible answers were “much better, better, equal, bad, very bad”: How do you feel compared to before the surgery? Evaluate your sleep in the last 3 nights? Judge your current appetite? How do you currently manage your personal hygiene? How do you manage going to toilet? How do you rate yourself when walking in the plane (independently of using crutches)? How do you rate yourself when climbing stairs (independently of using crutches)?

All patients furthermore received evaluation at a follow up after 12 months, while the following were recorded: physical examination, HHS, WOMAC, EQ-5D, EQ-VAS and PROMs.

### Statistical Analysis

For descriptive analysis, mean values and standard deviation are presented as continuous variables as well as absolute and relative frequencies for categorical variables. Comparisons of questionnaires over the course of time were performed by using a repeated-measures ANOVA. Mean differences with corresponding 95% confidence intervals (95%-CI) are presented as effect estimates. Multiple linear regression models were used to identify predictors for functional and quality of life outcomes after 4 weeks and 12 months. No adjustments of the significance level for multiple comparisons were performed. A *p*-value < 0.05 was considered statistically significant for all tests. All analyses were performed using SPSS 26.0 (IBM SPSS Statistics, IBM Corp., Armonk, NY, USA).

## 3. Results

**Figure 1:**
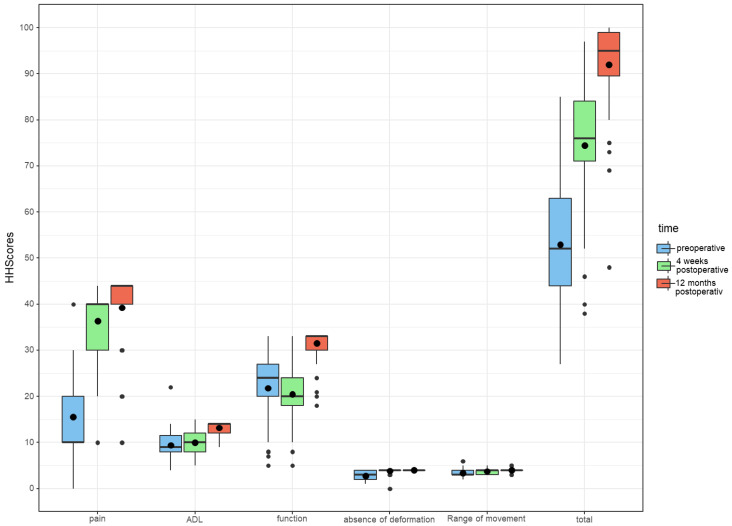
The Harris Hip score (HHS) and its subscales, preoperatively, 4 weeks and 12 months postoperatively. The HSS ranges between 0 and 100 and has 5 subscales: pain (0–44 points), activities of daily life (0–14 points), function (0–33 points), absence of deformity (0–4 points) and range of movement (0–5 points).

The Harris Hip Score (HHS) of the study collective showed a mean value of 53.0 (±13.0) preoperatively. At the first follow up after 4 weeks, the HHS improved to 74.5 (±12.9). At the follow up after 12 months, a further improvement to 92.0 (±9.4) was observed (Table 3). The mean difference from preoperatively to the follow up after 4 weeks was 21.5 (CI 17.5; 25.5) and to the follow up after 12 months 39.0 (CI 35.5; 42.5), which were both statistically significant (*p* < 0.001 (Figure 1).

The WOMAC score is divided into three subscales: pain, stiffness, physical function. The subscale of pain showed a mean improvement of 11.4 (±3.6) to 1.5 (±2.5) from preoperatively to 12 months postoperatively, the subscale stiffness of 4.69 (±1.74) to 1.08 (±1.36) and the subgroup physical function of 35.8 (±12.3) to 5.2 (±8.2) (Table 2). All value improvements were statistically significant (*p* < 0.001). The mean WOMAC total score was 53.1 (±15.7) preoperatively and improved until the follow up after one year to 8.5 (±11.8) and was statistically significant (*p* < 0.001).

The mean EQ-5D was 0.61 (±0.19) before surgery and improved to 0.94 (±0.10) one year after surgery (*p* < 0.001). The mean EQ-VAS score improved from preoperative 53.2 (±20.0) to 76.4 (±24.0) postoperative after one year (*p* < 0.001).

The correlation analysis of HADS, HADS-A score as well as HADS-D score showed no significant influence on WOMAC total score, WOMAC subscale pain, WOMAC subscale function, HHS after 4 weeks or one year and EQ-5D. Of the study collective, 11.5% showed borderline anxiety and 9.2% showed clinically relevant anxiety (HADS-A score). For the HADS-D score, 11.5% showed a borderline depression and 1.1% a clinically relevant depression (HADS-D score) (Table 4).

Neither age nor ASA score had a significant impact on the results of WOMAC total score, WOMAC subscale function, WOMAC subscale pain, HHS after 4 weeks and HHS after one year and EQ-5D. The WOMAC subscale score stiffness showed a significant correlation (*p* = 0.006) with age. Regarding preexisting conditions, a significant influence of diabetes mellitus type II on the result of HHS after 4 weeks and 12 months was shown (*p* = 0.003 and *p* = 0.020, respectively).

The PROMs (see Table 5) were analogically assessed 4 weeks and 12 months after surgery. After 4 weeks, 100% of the patients stated that the surgery was successful in their eyes, and almost 100% after 12 months stated that it was successful. Almost all patients would choose an enhanced recovery surgery (THA) again (98.9% and 97.9%, respectively). After 4 weeks, 69.4% of the patients felt “much better” in comparison to before the operation, and after one year, 84.4% of the patients felt “much better”. Already 4 weeks after fast-track THA, ≥ 80% of the patients stated that they were able to walk at ground level or climb stairs “well” or “very well”. During the study period, one patient received early revision surgery due to a postoperative seroma without evidence of infection. No other complications occurred.

## 4. Discussion

The present study evaluates the results of a newly introduced enhanced recovery concept in our department over a period of 12 months. The study results confirm a very high patient satisfaction after total hip arthroplasty by using an enhanced recovery concept. The interdisciplinary process optimization of all treatment pathways can lead to a shortening of hospital stay without affecting the quality and results of the treatment [4]. The HHS is a highly valid and reliable method of reporting outcomes after THA [21]. The minimum clinically important difference (MCID) is reported to be between four and ten points [22,23]. The postoperative mean values of HHS after 4 weeks and 12 months showed good to excellent results with a mean improvement of 21.0 points from preoperatively to 4 weeks follow up and 39.0 from preoperatively to 12 months follow up. Our results are consistent with the study of Larsen et al. [24], who investigated patient-related outcomes and the need for rehabilitation after fast-track THA in a prospective cohort study. Larsen et al. stated an HHS of 45.7 (±15.1) preoperatively, an HHS of 82.9 (± 13.1) 3 months after surgery and an HHS of 88.0 (± 15.1) 12 months after fast track hip arthroplasty. The study by Kutzner et al. [25], where the outcome of an enhanced recovery concept after THA was prospectively investigated, showed an HHS of 93.8 (± 8.0) 6 weeks after surgery and of 98.4. (± 3.0) 6 months after surgery. A significant improvement could be shown in HHS 12 and 18 months postoperatively after THA with a conventional or a fast track setup up, with no significant difference between both groups [26]. Fast-track THA gives equivalent functional outcomes compared to a traditional rehabilitation concept with simultaneous reduction of length of hospital stay and faster convalescence. However, this study was not designed to determine influencing factors on length of hospital stay, the focus of the study was to determine functional outcome and quality of life.

In our data, we have seen a significant influence of diabetes type II on the results of HHS after 4 weeks and 12 months. In the study by Jørgensen et al. [13], patients with diabetes type II had more comorbidities than nondiabetics. After adjusting for covariates, type II diabetics patients showed no longer hospital stay and increased readmission at 30 and 90 days. Our results are consistent with the study by Loth et al. [27] showing an impact of diabetes on the outcome after THA with lower preoperative and postoperative outcome scores compared to patients with no such conditions. 

We were able to show excellent WOMAC total scores, subscale of pain and subscale of function and a significant improvement in the 12 month follow up in comparison to preoperative values. There have been no studies found in the literature that compare preoperative to postoperative WOMAC score after THA in an enhanced recovery setup. Mariconda et al. [28] reported a high patient satisfaction after total hip arthroplasty and good results of WOMAC score in a long-term follow-up of 16 years.

The improvement of EQ-5D from preoperative to 12 months postoperative in our data was statistically significant (*p* < 0.001). After 12 months, the study collective showed a mean EQ-5D of 0.94, whereas an EQ-5D of 1.00 indicates full health. The EQ-5D index population norm for Germany in the age group of 55–64 is 0.881, and for the age group of 65–74 is 0.838 (European VAS value set) [29]. Preoperatively, the study population showed a lower EQ-5D compared to the index population norm, while 12 months postoperatively, the value increased to a higher level than the index population norm. Our results are consistent with the study of Larsen et al. [24], where EQ-5D showed a continued rise up to 12 months after surgery, and patients following fast track THA even reached a higher value than the population norm. The reason for the better values of the study population after 12 months compared to the population norm could be caused by a selection bias or by the applied technique of THA using an enhanced recovery concept itself. 

The hospital anxiety and depression scale is a valid and reliable tool. In our study, no influence of anxiety or depression on the outcome (HHS, WOMAC total, WOMAC subscale pain, WOMAC subscale function or EQ-5D) after enhanced recovery THA could be found. Previous studies showed that a higher preoperative anxiety score was predictive of residual pain after THA and TKA in general [30]. In a systematic review, post-operative pain was reported in 9% of patients after THA in the follow-up [31]. Improvements in pain and physical function after lower extremity total joint arthroplasty were associated with improvement in depression and anxiety symptoms [32]. After having had an acute pain experience, some patients are unable to resume their daily levels of activity and tend to regard pain as a threat [33]. This could lead to an automatic avoidance of harmful stimuli to reduce the risk of feeling pain [34] and to avoiding feared movement and activities. Since the fast-track concept involves initial mobilization 2–3 h after surgery, our consideration was that a patient with an anxious personality or depression might show a higher pain level, a poorer function and a poorer outcome. We assume that the missing correlation is caused by the small patient collective. Another consideration might be that preoperative patient information and education as well as gait training within the enhanced recovery concept has a positive impact on the patient’s anxiety and depression by reducing worries and fears before surgery.

In addition to the WOMAC, EQ-5D, HADS and HHS, we added subjective PROMs (Table 4) with partly “new” questions. Patient centeredness was defined by the WHO as a fundamental characteristic of the quality of healthcare [35]. PROMs evaluate the perspective of the patient and present outcomes that matter more to patients such as the impact on usual activities and self-care [36]. Measuring PROMs after arthroplasty surgery can be a key tool to improve healthcare quality. By asking the questions “Was the operation successful in your eyes?”, “Would you perform the surgery (THA) again?” and “How do you feel compared to before surgery?”, we wanted to check whether the patient’s expectations were fulfilled in the early postoperative course after 4 weeks and after 12 months. Almost 100% of the patients said that the surgery was rated successful and that they would perform enhanced recovery surgery again. More than 2/3 felt “much better” or “better” in the early postoperative course compared to preoperatively, even more than ¾ after 12 months. This results in a high level of patient satisfaction and could help surgeons and patients considering decision quality.

This study has several limitations. Firstly is the retrospective and record-based study design and the inclusion of a relatively small group of patients. Secondly, it is a newly established procedure in our department, which might have led to selection bias with a consecutive influence on the results. Furthermore, the maximum follow-up period in our study collective is limited to 12 months, so long-term results are not available. There was no direct comparison between patients with inpatient rehabilitation or outpatient rehabilitation, which could have led to a further bias. Regarding comorbidities, no power analysis was performed because it was a retrospective study design. Final conclusions can not be drawn. Further prospective long-term studies, possibly in a randomized controlled study design, should be undertaken to measure and conclude functional outcome and quality of life improvements after fast-track THA.

## 5. Conclusions

In the present study, patients participating in an enhanced recovery concept showed a high patient satisfaction after total cementless hip arthroplasty. During a follow-up of 4 weeks and 12 months, pain scores decreased and function scores increased to excellent values. Comorbidities like diabetes type II can have an impact on outcome after ERAS-THA. PROMs showed a continuous improvement over a follow-up of 12 months after the surgical procedure. PROMs reported in this study can help patients and surgeons to modify expectations of the surgery and to improve patient satisfaction.

## Figures and Tables

**Table 1:** Contents of enhanced recovery concept.

Preoperative checklist	Important information is already requested and documented during consultation: - Comorbidities - Allergies - Medication - Home care In case of multiple comorbidities anesthesiologic/internal presentation
Preoperative preparations	Information and preparation day approx. one week before surgery, detailed multidisciplinary lecture (anesthesia, surgery, physiotherapy) gait training with crutches
Surgery	Application of NSAID before surgerySpinal anesthesia Pre- and intraoperativelocal infiltration analgesia (LIA) and tranexamic acid (TXA)Anterolateral approach (Microhip)No wound drains
Intensive physiotherapeutic care	First mobilization 2–3h after surgery with full weight bearingPhysiotherapeutic treatment 2×/day with gait training and exercises Instruction to exercise circuit
Pain management	Recommendations within the WHO analgesic ladder
Discharge criteria	Absence of wound problemsSatisfactory pain control Knowledge of restrictions and being able to walk safely at ground level and stairs

**Table 2:** General and demographic data.

*n* (total)		109
Gender	Male	70 (64.2%)
	Female	39 (35.8%)
Age (in years)		62.1 (±10.5)
Side of operation	Right	*n* = 57 (52.3%)
	Left	*n* = 52 (47.7%)
Body Mass index (kg/m^2^)		28.1 (±4.45)
ASA	1	*n* = 30 (27.5%)
	2	*n* = 72 (66.1%)
	3	*n* = 7 (6.4%)
	4	*n* = 0 (0%)
ECM	0	*n* = 41 (37.6%)
	1	*n* = 40 (36.7%)
	2	*n* = 20 (18.3%)
	>3	*n* = 8 (7.3%)
Coronary heart disease		*n* = 14 (12.8%)
Arterial hypertension		*n* = 40 (36.7%)
Diabetes mellitus type II		*n* = 9 (8.3%)

ASA: American Society of Anesthesiologists, ECM: Elixhauser Comorbidity Method. Categorial variables are given in absolute numbers/percent and continuous variables in mean values/±standard deviation.

**Table 3:** PROM scores preoperatively, 4 weeks and 12 months after surgery.

	Preoperative	Follow Up: 4 Weeks	Follow Up 12 Months
Harris Hip score	52.95 ± 12.98	74.47 ± 12.92	91.99 ± 9.40
WOMAC total score	53.07 ± 15.73		8.52 ± 11.81
WOMAC subscale pain	11.36 ± 3.60		1.51 ± 2.52
WOMAC subscale function	35.79 ± 12.28		5.72 ± 8.19
WOMAC subscale stiffness	4.69 ± 1.74		1.08 ± 1.34
EQ-5D	0.61 ± 0.19		0.94 ± 0.10
EQ-VAS	53.25 ± 19.97		76.36 ± 24.03

Mean values/±standard deviation.

**Table 4:** Hospital Anxiety and Depression Scale (HADS).

HADS Score	≤7 Points	8–10 Points	≥11 Points
HADS-A	79.3%	11.5%	9.2%
HADS-D	87.4%	11.5%	1.1%

HADS-A represents the domain of anxiety and HADS-D the domain of depression.

**Table 5:** PROMs after a follow up of 4 weeks and 12 months after surgery.

PROM	Follow Up 4 Weeks	Follow Up 12 Months
Was the operation successful in your eyes?	Yes = 100%	Yes = 96.9%
	No = 0%	No = 3.1%
Would you perform the surgery (THA) again?	Yes = 98.9%	Yes = 97.9%
	No = 1.1%	No = 2.1%
How do you feel compared to before surgery?	Much better = 69.4%	Much better = 84.4%
	Better = 27.6%	Better = 12.5%
	Equal = 3.0%	Equal = 2.1%
	Bad = 0%	Bad = 1.0%
Evaluate your sleep in the last 3 nights?	Very good = 14.4%	Very good = 43.3%
	Good = 49.5%	Good = 45.4%
	Moderate = 24.7%	Moderate = 7.2%
	Bad = 9.3%	Bad = 3.1%
	Very bad = 2.1%	Very bad = 1.0%
Judge your current appetite?	Very good = 45.4%	Very good = 68.0%
	Good = 50.5%	Good = 30.9%
	Moderate = 4.1%	Moderate = 1.0%
How do you currently manage your personal hygiene?	Very good = 44.8%	Very good = 75.5%
	Good = 47.9%	Good = 24.5%
	Moderate = 7.3%	Moderate = 0%
How do you manage going to toilet?	Very good = 59.8%	Very good = 85.7%
	Good = 37.1%	Good = 14.3%
	Moderate = 3.1%	Moderate = 0%
How do you rate yourself when walking in the plane?	Very good = 27.8%	Very good = 61.2%
	Good = 58.8%	Good = 33.7%
	Moderate = 13.4%	Moderate = 3.1%
	Bad = 0%	Bad =2.0%
How do you rate yourself when climbing stairs?	Very good = 25.8%	Very good = 58.2%
	Good = 55.7%	Good = 34.7%
	Moderate = 16.5%	Moderate = 4.1%
	Bad = 2.1%	Bad = 3.1%

Indication of the answer in percent of patients.

## Data Availability

Not applicable.
